# Albumin–Methotrexate Prodrug Analogues That Undergo Intracellular Reactivation Following Entrance into Cancerous Glioma Cells

**DOI:** 10.3390/pharmaceutics14010071

**Published:** 2021-12-28

**Authors:** Itzik Cooper, Michal Schnaider-Beeri, Mati Fridkin, Yoram Shechter

**Affiliations:** 1 The Joseph Sagol Neuroscience Center, Sheba Medical Center, Tel Hashomer, Ramat Gan 52621, Israel; michal.beeri@sheba.health.gov.il; 2 School of Psychology, Reichman University, Herzliya 4610101, Israel; 3 The Nehemia Rubin Excellence in Biomedical Research—The TELEM Program, Sheba Medical Center, Tel Hashomer, Ramat Gan 52621, Israel; 4 Department of Psychiatry, Mount Sinai School of Medicine, New York, NY 10029, USA; 5 Department of Organic Chemistry, The Weizmann Institute of Science, Rehovot 76100, Israel; mati.fridkin@weizmann.ac.il; 6 Department of Biomolecular Sciences, The Weizmann Institute of Science, Rehovot 76100, Israel; yoram.shechter@weizmann.ac.il

**Keywords:** cancer, albumin, disulfide, glioma, prodrug, conjugate

## Abstract

A family of monomodified bovine serum albumin (BSA) linked to methotrexate (MTX) through a variety of spacers was prepared. All analogues were found to be prodrugs having low MTX-inhibitory potencies toward dihydrofolate reductase in a cell-free system. The optimal conjugates regenerated their antiproliferative efficacies following entrance into cancerous glioma cell lines and were significantly superior to MTX in an insensitive glioma cell line. A BSA–MTX conjugate linked through a simple ethylene chain spacer, containing a single peptide bond located 8.7 Å distal to the protein back bone, and apart from the covalently linked MTX by about 12 Å, was most effective. The inclusion of an additional disulfide bond in the spacer neither enhanced nor reduced the killing potency of this analogue. Disrupting the native structure of the carrier protein in the conjugates significantly reduced their antiproliferative activity. In conclusion, we have engineered BSA–MTX prodrug analogues which undergo intracellular reactivation and facilitate antiproliferative activities following their entrance into glioma cells.

## 1. Introduction

Methotrexate (MTX) is an antiproliferative and immunosuppressive agent widely and effectively used against a broad spectrum of diseases, primarily cancerous tumors but also certain autoimmune diseases such as psoriasis and rheumatoid arthritis [1]. It facilitates its antiproliferative effects by inhibiting dihydrofolate-reductase (DHFR), a ubiquitous enzyme that catalyzes the reduction of folate to dihydrofolate (DHF) and then to tetrahydrofolate. The latter is a cofactor for de novo synthesis of thymidylate, purine, and glycine [2].

As with most antimetabolites, MTX is only partially selective for tumor cells and is toxic to all rapidly dividing normal cells, such as those of the intestinal epithelium and bone marrow [3]. Although relatively less toxic compared with other therapeutic agents, it is not devoid of side effects. In addition, a variety of tumor cells develop resistance to MTX upon treatment with agents, affecting several mechanistic pathways, including impaired transport of MTX into cells [4,5]. Solving these deficiencies and increasing MTX selectivity toward inflamed, diseased, or malignant tissues is therefore of primary clinical significance.

One of the approaches undertaken in recent years as an attempt to increase selectivity of chemotherapeutic agents was to link them covalently to macromolecules such as albumin. Extravasation of albumin is upregulated about six-fold in inflamed, diseased, and malignant tissues [6,7]. If the covalently-linked MTX has low antiproliferative activity, this would create a rather profound clinical advantage, provided that the conjugate undergoes efficient intracellular reactivation following entrance into tumorigenic cells. The amide bond of MTX linked to the ε-amino group of lysine is chemically and enzymatically stable [8], and reactivation appears to be dependent on extensive intracellular proteolysis, as only MTX linked to short peptide fragments through its γ-carboxylate moiety regain significant antiproliferative efficacy (this study). Similarly, binding MTX to other macromolecules, such as anti-TNFα antibodies, might be clinically beneficial in autoimmune and inflammatory diseases such as arthritis, for example, where MTX is administrated at low dosages separately from anti-TNFα antibodies. Generating such an antibody–MTX conjugate may reduce side effects and improve efficacy as well as provide better treatment tolerance.

We thus initially searched for a chemical procedure to covalently link MTX to amino-containing molecules through the γ-carboxylate moiety of MTX. The linkage of such molecules to the α-carboxylate of MTX was documented to yield inactive analogues [9,10]. Secondly, we examined the possibility of introducing MTX to albumin through spacers containing disulfide bonds. Malignant tissues have elevated de novo synthesis of glutathione [11,12,13], and we wondered whether this would lead to increased selectivity and enhanced reduction-dependent release of active MTX in tumorigenic cells.

In this study, we describe our synthetic procedures for obtaining a variety of BSA–MTX conjugates, their cell-free DHFR-inhibitory potencies in the absence and in the presence of glutathione or cysteine, and their antiproliferative efficacies in several lines of cancerous cells relative to that of methotrexate. Two glioma cell lines are investigated here, for the first time, in the context of albumin conjugates to align with other research in our lab, in which new compounds and methods that enable blood–brain barrier (BBB) opening, and thus, the entrance of large therapeutic molecules such as albumin are being developed [14,15,16,17,18].

## 2. Experimental Procedures

### 2.1. Materials

Methotrexate, bovine serum albumin, cystamine dihydrochloride, hexamethyl diamine-dihydrochloride, dihydrofolate (DHF), *N*,*N*′dicyclohexylcarbodiimide (DCC), β-nicotinamide adenosine dinucleotide 2′ phosphate (NADPH), reduced glutathione (GSH) oxidized glutathione (GSSG), and l-cysteine were purchased from Sigma (St. Louis, MO, USA). Methotrexate linked to a peptide (MTX–AYGRKKRRQRRR) was synthesized by the manual conventional solid-phase synthesis. An Fmoc (N-9-flurenylmethoxy carbonyl) strategy was employed through the peptide chain assembly. All other materials used in this study were of analytical grade.

### 2.2. Synthesis of MTX–Anhydride

Methotrexate (45.4 mg, 100 μM) dissolved in 0.7 mL dimethylformamide (DMF) and 95 μL from a solution of 1 M DCC in DMF (95 μM) was added. The reaction was carried out for 3 h at 25 °C. Dicyclohexylurea was filtered out. The MTX–anhydride formed was kept at 4 °C until used.

### 2.3. Synthesis of MTX–Dicystamine [MTX–CO^γ^NH–(CH_2_)_2_–S–S–(CH_2_)_2_–NH_2_]

Dicystamine di-HCl (100 μM) was dissolved in 0.7 mL DMF, neutralized with DIPEA, and combined with the solution of MTX–anhydride (150 μM in 0.7 mL DMF). The reaction mixture was stirred for 40 min, precipitated with ether, washed three times with ether, and desiccated. The solid material was suspended in H_2_O and centrifuged. This procedure, which removes residual dicystamine and unreacted MTX, was repeated five times. The solid material was then lyophilized. MTX–CO^γ^NH–(CH_2_)_3_–S–S–(CH_2_)_2_–NH_2_ was obtained in 60% yield. It has a molar extinction coefficient similar to that of MTX (ε_305nm_ = 22,700 and ε_372nm_ = 7200). Calculated ESMS = 588 Da, found ESMS for M − H = 587.26 Da.

### 2.4. Synthesis of MTX–Hexamethyl-Amine and MTX–Glutathione

These derivatives were synthesized by the same procedure applied for MTX–dicystamine except that dicystamine was replaced with hexamethyldiamine-di-HCl or with GSSG. MTX–hexamethyl-amine [MTX–CO^γ^NH(CH_2_)_6_–NH_2_] and MTX–glutathione [MTX–CO^γ^NH–GSSG] were obtained in 28 and 37% yields, respectively. Calculated ESMS for MTX–hexamethylamine is 552 Da, found for M + H = 553.41 Da, and M − H = 551.38 Da. Calculated ESMS for MTX–GSSG is 1048 Da, found M + H = 1049.4, and M − H = 1047.51.

### 2.5. Preparation of BSA–MTX Conjugates

MTX–dicystamine (4 μM) was dissolved in 50 μL DMF and combined with a solution of MAL–(CH_2_)_3_–COOSu (3 μM in 50 µL DMF). Following one hour of incubation, this reaction mixture was added in 9 aliquots (10 μL each) over a period of one hour to a stirred solution of BSA (3.0 mL, 70 mg/mL in 0.1 M HEPES buffer (pH 7.4). The reaction was carried out at 0 °C. The derivative thus obtained was dialyzed over a period of 3 days against H_2_O, 0.03 M Na_2_CO_3_ (pH 10.3), and H_2_O, and then lyophilized. BSA–S–S–MTX contains 0.7 ± 0.05 M MTX per mole BSA as determined by its absorbance at 372 nm using ε_372nm_ = 7200. The protein concentration was determined by amino acid analysis following acid hydrolysis of a measured aliquot, calculated according to valine (36 residues) isoleucine (14 residues) and lysine (59 residues).

BSA–(CH_2_)_6_–MTX and BSA–GSSG–MTX were prepared essentially by the same procedure applied for BSA–S–S–MTX (previous section), except that MAL–(CH_2_)_3_–COOSu was linked to either MTX–hexamethylamine or to MTX–GSSG for obtaining either BSA–(CH_2_)_6_–MTX or BSA–GSSG–MTX, respectively. Analogues which contained covalently linked MTX in the range of 0.6 to 0.8 M MTX per mole of BSA were subjected to further studies.

### 2.6. Cleavage of BSA–(CH_2_)_6_–MTX with Cyanogen Bromide

BSA–(CH_2_)_6_–MTX (40 mg) was dissolved in 1.0 mL 70% formic acid. Solid cyanogen bromide (20 mg) was then added and incubated for 24 h at 25 °C. The reaction mixture was evaporated to dryness, dissolved in 1.0 mL H_2_O, dialyzed for two days against H_2_O, and lyophilized. Derivatives concentration and MTX content was obtained by quantitative amino-acid analysis and by the absorbance at 372 nm, as described in detail in the previous section.

### 2.7. Preparation of HSA(MTX)_40_

HSA (67 mg, 1 μM) was dissolved in 10 mL of 0.1 M HEPES buffer pH 7.4, and MTX–anhydride (100 μM in 0.7 mL DMF) was added in 7 aliquots (100 μL each) over a period of 2 h at 0 °C to the stirred solution of the protein. The derivative thus obtained was dialyzed over a period of 3 days against H_2_O, 0.03 M Na_2_CO_3_ (pH 10.3), and H_2_O, and then lyophilized. HSA-(MTX)_40_ contains 40 ± 3 M MTX per mole of HSA as determined by the absorbance at 372 nm (ε_372nm_ = 7200). The protein content was determined by quantitative amino acid analysis following acid hydrolysis of a measured aliquot, calculated according to glycine (12 residues), alanine (62 residues), and valine (41 residues).

### 2.8. Partial Purification of DHFR from Chicken Liver

This was carried out by the procedure of Kaufman and Kemerer [19] with some modifications: Fresh chicken liver (20 g) was cut into pieces, homogenized in 0.1 M MnCl_2_ and centrifuged. The supernatant was brought to pH 5–6 with HCl, and ZnSO_4_ was added gradually to obtain a final concentration of 20 mM. The pH was elevated to pH 7.5–8 by the addition of solid NaHCO_3_. The precipitate thus formed (about 87% of total protein) was removed by centrifugation. The proteins that remained in the supernatant were precipitated with 4.54 M ammonium sulfate. The precipitate was then dissolved in 50 mM Tris-HCl (pH 7.5) containing 0.1 M KCl and dialyzed for two days against the same buffer, and then frozen in aliquots at −80 °C until used. Partially purified DHFR prepared by this procedure has a specific activity of about 0.05 units/mg.

### 2.9. Cell-Free Enzymatic Assay for DHFR

The assay was based on DHFR-dependent reduction of dihydrofolate to tetrahydrofolate. In this process, NADPH is oxidized to NADP, and the extent of the oxidation is monitored by the decrease in absorbance at 340 nm [20]. Tubes containing 1.0 mL each of 0.05 M Tris-HCl buffer (pH 7.5) 0.5 M KCl, 0.137 mM NADPH, and 0.187 mM DHF were incubated for 1 h at 25 °C in the absence and the presence of partially purified DHFR (35 µg/mL) and increasing concentrations of MTX or its derivatives. The absorbance at 340 nm was then monitored. In a typical assay, absorbance at 340 nm amounted to 1.7 ± 0.06 and 0.65 ± 0.06 in the absence and the presence of DHFR. MTX inhibits DHFR with an IC_50_ value of 55 nM. A methotrexate derivative yielding an IC_50_ value of 0.55 μM was considered as having 10% of the inhibitory potency of the native folic acid antagonist.

### 2.10. Maleimide–Thiol Exchange Assay

BSA–S–MAL–(CH_2_)_3_–CONH–MTX (0.2 mM) was dissolved in 1.0 mL 0.1 M HEPES, pH 7.3, and transferred to a tube containing 7.5 mg GSH corresponding to 24 µM which are 100 molar excess over the conjugate. The solution was incubated for 7 h at 37 °C, followed by extensive dialysis and lyophilization. Absorbance was read at 372 nm.

### 2.11. Cancer Cell Lines

In this study, we used two glioma cell lines: (i) CNS-1; rat glioma; (ii) U251; human glioma. Cells were a generous gift from Dr. Yael Mardor from the Sheba Medical Center, Israel.

### 2.12. Growth Inhibition Effects of BSA–MTX Analogues

Cancer cell lines were grown in monoculture in 96-well plates in RPMI (CNS-1 cells) or DMEM containing 10% fetal bovine serum, 2 mM l-glutamine, penicillin (100 units/mL), and streptomycin (0.1 mg/mL) under humidified atmosphere containing 5% CO_2_. Following 24 h or 48 h, MTX or BSA–MTX analogues were added to each plate to give a final MTX or HSA–MTX analogue concentration ranging from 1 nM to 10 µM. Cell viability was measured after 48 h using a standard MTT (3-(4,5-dimethylthiazol-2-yl)-2,5-diphenyltetrazolium bromide) assay, as described before [21].

### 2.13. Statistical Analysis

Statistical analysis was performed using the Prism (GraphPad) 6 software. Data are presented as the means ± standard error of the mean (SEM). Differences between two groups were assessed by an unpaired t-test and among three or more groups by a one-way analysis of variance followed by Tukey′s multiple comparison test. A *p*-value of less than 0.05 was considered statistically significant.

## 3. Results

### 3.1. Searching for a Chemical Procedure to Obtain Active MTX Derivatives Following the Covalent Introduction of Amino-Containing Nucleophiles

Of the two carboxylates of MTX, the α carboxylate participating in binding to DHFR and its derivatization was documented to yield inactive analogues [9,10]. The γ carboxylate of MTX is more distal to the binding site of DHFR, and its derivatization can yield products with high or moderate affinity toward the enzyme [22,23]. In preliminary experiments, we found that the covalent linkage of MTX to the N-terminal position of a peptide (TAT, Table 1), synthesized by manual conventional peptide synthesis, yielded MTX–peptide having little MTX-inhibitory potency (IC_50_ = 1.0 ± 0.1 μM, ~5.5% of MTX, summarized in Table 1), suggesting that a peptide-bond formation by the routine carbodiimide solid-phase synthesis takes place predominantly through the α-amino side chain of MTX.

**Scheme 1:**
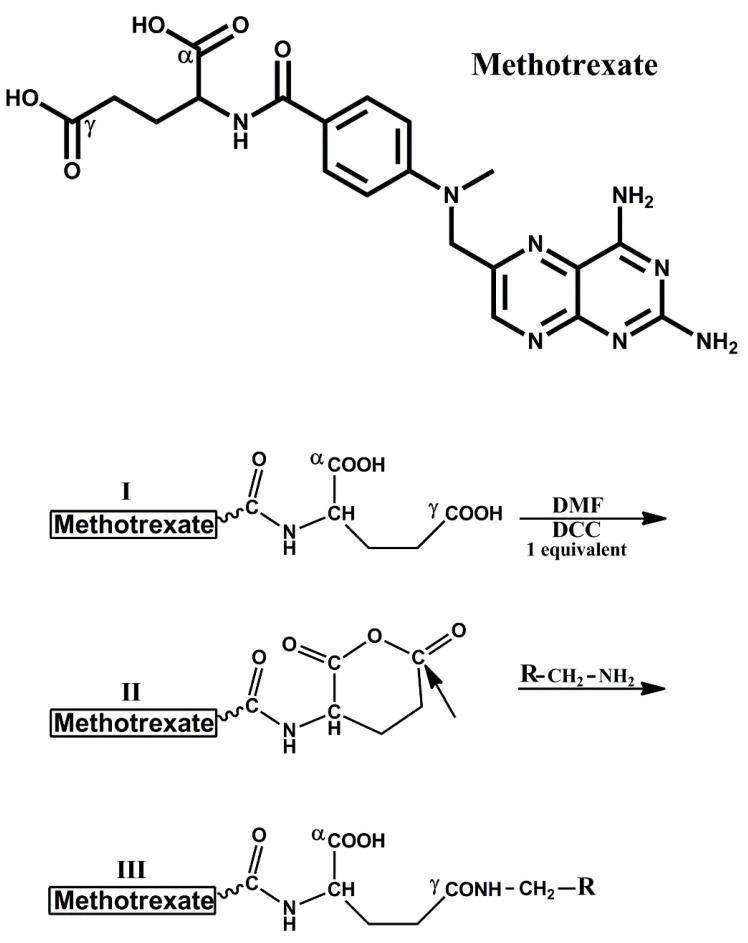
The schematic synthetic procedure used to link amino-containing nucleophiles to the γ-carboxylate moiety of methotrexate. Methotrexate is initially treated with one equivalent of dicyclohexylcarbodiimide (DCC) to obtain the mixed anhydride (II). Amino-containing nucleophiles such as R–CH_2_–NH_2_ react preferentially with the γ-carbon (indicated by the arrow) to obtain γ-carboxylate-substituted derivatives (III) owing to the higher pKa (pKa~4.1) of the γ-carboxylate, as opposed to that of the α-carboxylate (pKa~2.1). A detailed description of this procedure can be found in Methods.

**Figure 1:**
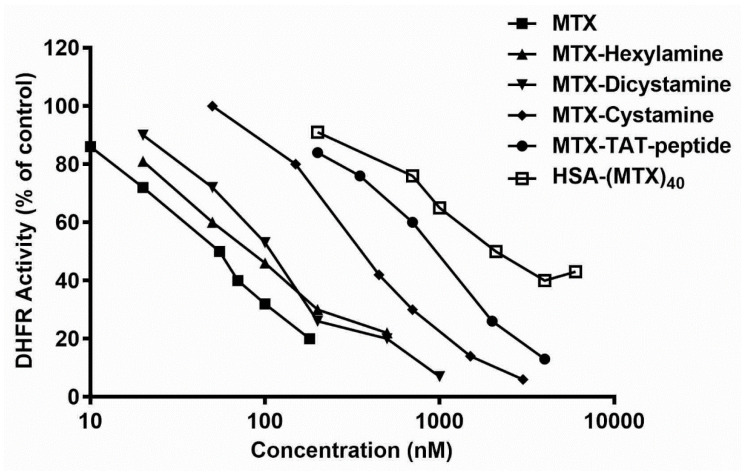
Inhibition of dihydrofolate reductase by MTX and MTX analogues. The indicated concentrations of MTX and its derivatives were analyzed for their potencies to inhibit the reduction of dihydrofolate to tetrahydrofolate by DHFR. In this process, NADPH is oxidized to NADP and the extent of this oxidation is monitored by the decrease in absorbance at 340 nm (experimental section). OD_340_ amounted to 1.7 ± 0.06 in the absence of the enzyme (taken as 100% inhibition) and to O.D_340_ = 0.65 ± 0.06 in its presence (taken as 100% activity).

Huang et al. investigated the reaction of aniline with α-amino-blocked l-glutamic acid anhydride. They found that this reaction yielded almost exclusively the γ-anilide isomer [24]. Based on that, we prepared MTX–anhydride by treating MTX with one equivalent of DCC in DMF. A stoichiometric amount of DCU was precipitated within a period of 0.5 h, indicating that MTX–anhydride was formed in a quantitative fashion under these experimental conditions (Scheme 1). The reaction of MTX–anhydride with low molecular-weight amino-containing nucleophiles (R–CH_2_–NH_2_) yielded analogues which preserved 16–69%, the DHFR inhibitory potency of MTX (IC_50_ values in the range of 0.08–0.34 μM, Table 1). Out of the five MTX derivatives studied, MTX–CONH–(CH_2_)_6_–NH_2_ was the most potent. It preserved 69% the potency of MTX (IC_50_ = 0.08 μM, Figure 1 and Table 1). Thus, linking amino-containing compounds up to a molecular weight of about 300 Da by this procedure yields fairly potent MTX derivatives. We next reacted human serum albumin (HSA) with MTX–anhydride obtaining HSA derivatives containing up to 40 M of MTX per mole of HSA linked to the amino side-chains of the protein (experimental procedure). HSA–(MTX)_40_ had only 2.6% the inhibitory potency of MTX (IC_50_ = 2.1 ± 0.2 μM, Figure 1 and Table 1), indicating that MTX linked to a macromolecule such as albumin through its γ-carboxylate moieties is, in principle, a prodrug, provided that such conjugates are capable of undergoing reactivation following entrance into tumorigenic cells.

### 3.2. Preparing BSA–MTX Prodrug Analogues

**Scheme 2:**
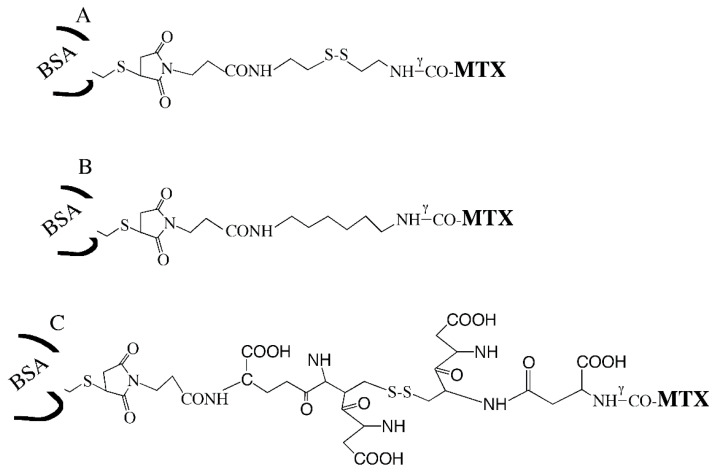
Schematic presentation of the three monomodified analogues of BSA–MTX. (**A**) BSA–S–S–MTX, (**B**) BSA–(CH_2_)_6_–NH–^γ^–CO–MTX, and (**C**) BSA–GSSG–NH–^γ^–CO–MTX.

Bovine serum albumin has a single cysteinyl moiety (cysteine 34), enabling introduction of MTX in a monomodified fashion, through a spacer maleimide (MAL) molecule linked to MTX through its γ-carboxylate moiety (experimental procedure). Three such analogues were prepared: BSA–S–S–MTX; [BSA–S–MAL–(CH_2_)_3_–CONH–(CH_2_)_2_–S–S–(CH_2_)_2_–NH^γ^CO–MTX], BSA–GSSG–MTX; [BSA–S–MAL–(CH_2_)_3_–CO–NH–GSSG–NH^γ^CO–MTX] and BSA–(CH_2_)_6_–NH^γ^CO–MTX; [BSA–S–MAL–(CH_2_)_3_–CONH–(CH_2_)_6_–NH^γ^CO–MTX] (Scheme 2). In the first two analogues, the spacer contains a disulfide bond. This was designed to evaluate whether the cells are equipped with cytosolic or endocytic disulfide bond-breaking machinery, and whether such activity would elevate the antiproliferative efficacy of those disulfide-containing analogues.

### 3.3. BSA–MTX Analogues Are Prodrugs

**Figure 2:**
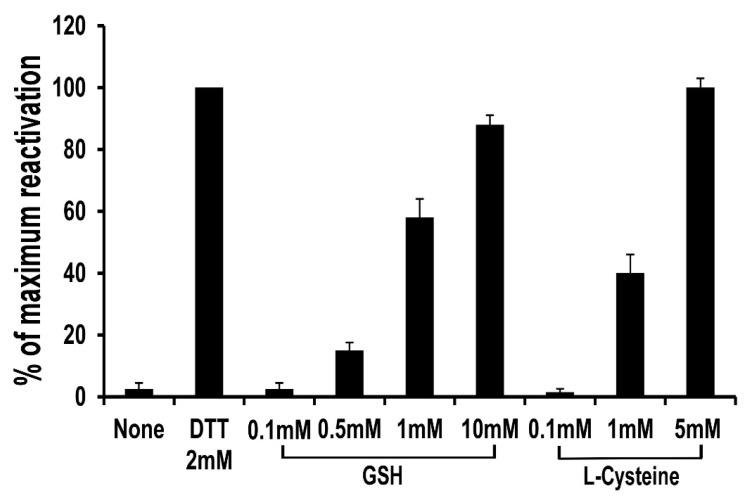
Reactivation of BSA–GSSG–MTX by reduced glutathione and l-cysteine. A solution of BSA–GSSG–MTX (50 μM in 0.1 M HEPES buffer, pH 7.4, 130 mM NaCl) was divided into plastic tubes (1.0 mL/tube) and incubated for 2 h at 25 °C in the absence and the presence of 2 mM DTT and the indicated concentrations of either GSH or l-cysteine. Samples were then analyzed for their DHFR inhibitory potencies and the IC_50_ values were determined. Results are expressed as percent of maximal reactivation (obtained by preincubation with 2 mM DTT). Each point is the arithmetic mean of *n* = 4 tubes ± SEM.

All three BSA–MTX analogues prepared had 2% to 7% the potency of MTX, to inhibit DHFR in a cell-free assay (summarized in Table 2). The disulfide-containing derivatives undergo 5- to 7-fold reactivation in the presence of 2mM dithioerythritol (Table 2). These analogues also undergo reactivation by reduced glutathione (GSH) and by l-cysteine (IC_50_-value 0.8–1.2 mM for both, Figure 2). Glutathione and l-cysteine do not reactivate BSA–GSSG–MTX or BSA–S–S–MTX (not shown) at concentrations of 100 μM or lower (Figure 2). Thus, all three analogues expect to be systemically inactive, and therefore are nontoxic in body fluids, as those are devoid from significant amounts of disulfide-reducing agents such as GSH [12].

### 3.4. Lack of Maleimide–Thiol Exchange of BSA–(CH_2_)_6_–MTX following Treatment with Reduced Glutathione (GSH)

Shen et al. [25] have demonstrated that SH-containing proteins, linked via maleimide to drugs, can undergo maleimide–thiol exchange if exposed to cysteine or to glutathione. To examine if such exchange takes place with BSA–S–MAL–(CH_2_)_3_–CONH–(CH_2_)_6_–NHCO–MTX, we incubated this derivative with 100 molar excess of GSH for a period of 7 h at pH 7.3, 37 °C. The conjugate was then dialyzed, lyophilized, and examined for its absorbance at 372 nm. As can be seen in Table 3, BSA–S–MAL–(CH_2_)_3_–CONH–MTX did not undergo significant maleimide–thiol exchange following prolonged incubation with 100-fold molar excess of GSH. This suggests that the maleimide linked to cysteine 34 of BSA is not exposed to the external medium.

### 3.5. Selecting MTX-Sensitive and MTX-Insensitive Glioma Cell Lines for Studying the Antiproliferative Effects of BSA–MTX Analogues

**Figure 3:**
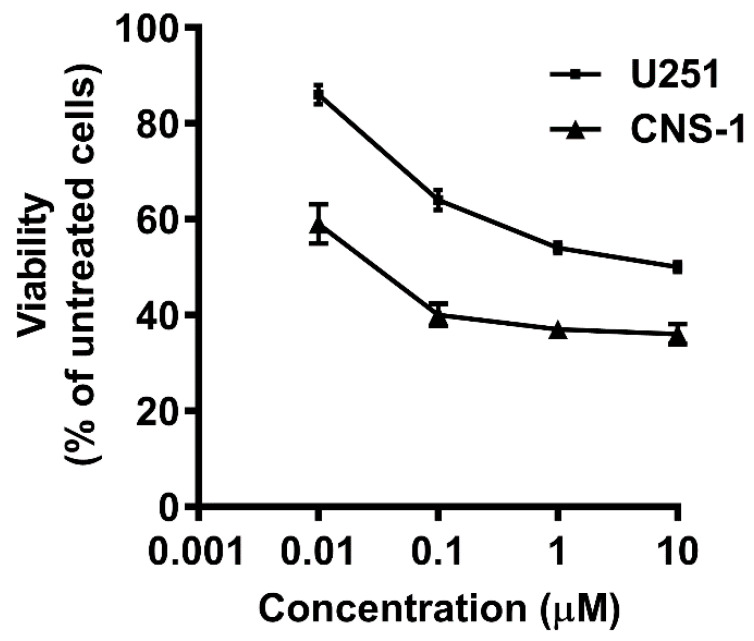
Viability of CNS-1 and U251 towards MTX. CNS-1 and U251 glioma cells were seeded in 96-well plates and MTX was applied a day later at the indicated concentrations. MTT assay was performed after 48 h. Viability is presented as mean ± SEM of each group in comparison to untreated cells. *n* ≥ 20 from at least six different experiments. There is a significant difference of *p* < 0.01 between the two cell lines in all concentrations measured.

The documented inhibitory effects of MTX in a variety of glioma cell lines has an average EC_50_ of 2.4 µM with a large variation among different lines [26]. Following screening, we selected two glioma cell lines which differ significantly in their response to MTX. The first is an MTX-sensitive rat glioma cell line (CNS-1) with EC_50_ (the effective concentration needed to obtain 50% of viable cells relative to untreated cells) of ~0.07 µM and 64% maximal effect (i.e., 36% viability in comparison to nontreated cells) within 48 h. The second cell line, the human glioma U251, is relatively insensitive to MTX. It has EC_50_ of ~10 µM which is also the concentration needed to obtain a 49% maximum effect within 48 h of treatment (Figure 3).

### 3.6. Comparison of the Antiproliferative Potencies of the BSA–MTX Conjugates in the Insensitive and Sensitive Glioma Cell Lines

**Figure 4:**
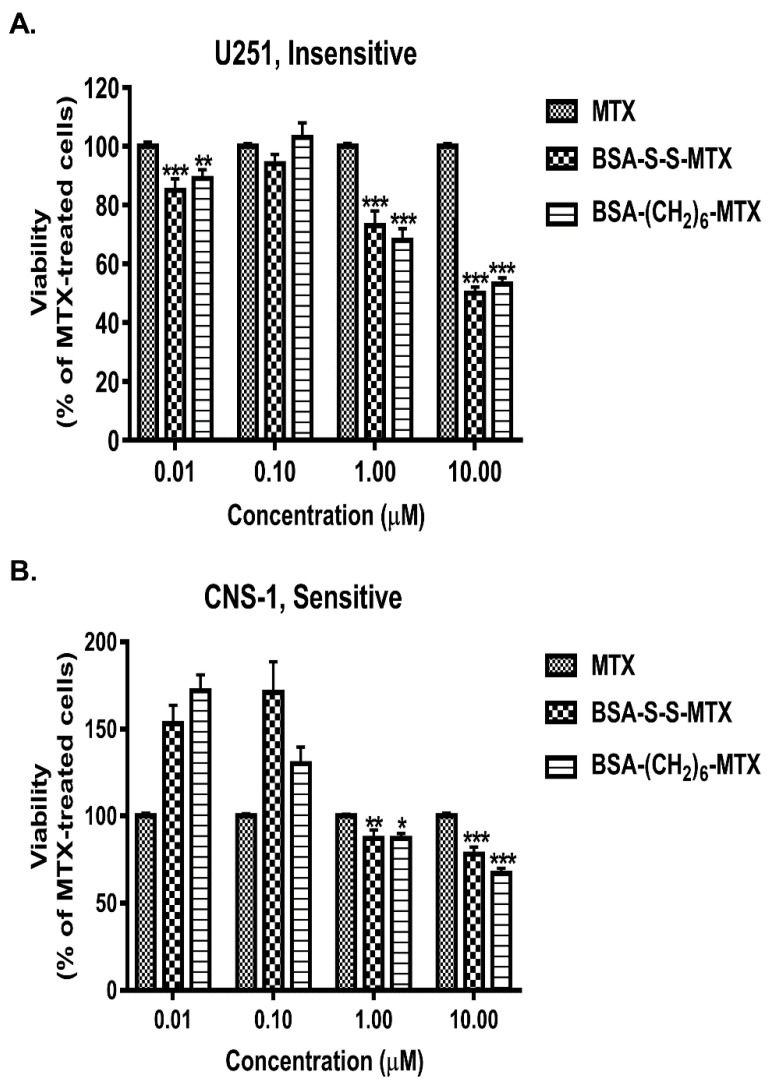
Viability of CNS-1 and U251 towards BSA–MTX derivatives. U251 (**A**) and CNS-1 (**B**) glioma cells were seeded in 96-well plates, and a day later, BSA–MTX derivatives were added at the concentrations shown. MTT assay was performed after 48 h to measure the viability of the cells. Viability of each conjugate was normalized to the viability of the cells treated with free MTX at each concentration. Data are presented as average mean ± SEM of each group in comparison to MTX-treated cells. *n* ≥ 20 from at least three different experiments. * *p* < 0.05, ** *p* < 0.01, *** *p* < 0.001 vs. MTX-treated cells.

Both BSA–MTX conjugates, BSA–S–S–MTX and BSA–(CH_2_)_6_–MTX, were found to be significantly more potent in the MTX-insensitive glioma cell line (Figure 4). As expected, those BSA–MTX analogues were less potent than MTX in the MTX-sensitive cell line. Nevertheless, at 10 µM (and to a lesser extent also at 1 µM), the BSA–MTX conjugates were significantly more inhibitory than MTX (Figure 4B).

### 3.7. Analyzing the Antiproliferative Potencies of Albumin–MTX Conjugates That Lost the Native Three-Dimensional Structure of the Protein

**Figure 5:**
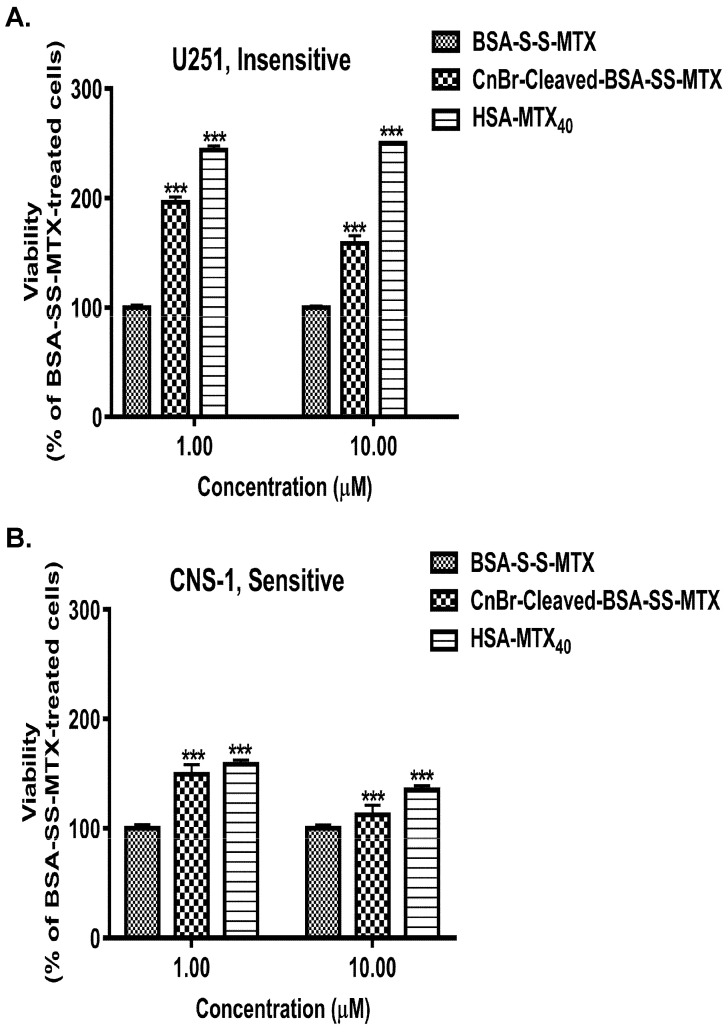
Viability of CNS-1 and U251 towards HSA–(MTX)_40_ and CNBr-cleaved BSA–S–S–MTX. U251 (**A**) and CNS-1 (**B**) glioma cells were seeded in 96-well plates, and a day later, derivatives were added at the concentrations mentioned in the Figure. MTT assay was performed after 48 h to measure the viability of the cells. Viability of each conjugate was normalized to the viability of the cells treated with BSA–S–S–MTX at each concentration. Data are presented as average mean ± SEM of each group in comparison to BSA–S–S–MTX–treated cells. *n* ≥ 12 from at least four different experiments. *** *p* < 0.001 vs. BSA–S–S–MTX–treated cells.

We prepared two analogues of albumin–MTX in which the native structure of the carrier protein was disrupted, either by cleaving it at four sites with cyanogen bromide (CNBr-cleaved BSA–S–S–MTX) or derivatizing several of its amino side chains with MTX (HSA–MTX_40_). Both analogues had negligible potency to inhibit DHFR in the cell-free system (Table 2). We next determined whether it is possible to regenerate activity in the glioma cell lines. Both analogues lost a significant amount of their antiproliferative potency and were significantly less effective in reducing the viability of the glioma cells in comparison to BSA–S–S–MTX (Figure 5). This was seen with both glioma cell lines in the concentration range of 1–10 µM at which BSA–S–S–MTX and BSA–(CH_2_)_6_–MTX are highly potent. Thus, it appears that preservation of the native structure of the carrier protein is required for either entry into those cell lines and/or obtaining the appropriate fragmentation requiring to turn those “silent” analogues into cytotoxic-active species.

The inclusion of a disulfide bond in the spacer connecting MTX to BSA does not elevate the antiproliferative effect of the conjugate. As malignant tissues have elevated intracellular GSH, reaching concentrations as high as 10 mM [27,28] while the circulatory system possesses low reductive potency [29], it was logical to assume that BSA–MTX conjugates containing a disulfide bond will have increased selectivity and enhanced killing potency towards cancerous cell lines in general. We found that BSA–S–S–MTX is slightly less potent than BSA–(CH_2_)_6_–MTX, both in the sensitive and the insensitive glioma cell lines (Figure 4). This is in spite of the fact that GSH, at a concentration of 1 mM or higher, reactivated this conjugate, as shown by its efficacy to inhibit DHFR in the cell-free state (Figure 2). Thus, it suggests that the conjugate is not exposed at all to the cytosolic glutathione following internalization.

## 4. Discussion

In this study, we have engineered, synthesized, and studied conjugates of bovine-serum albumin linked to methotrexate (BSA–MTX conjugates). Those were found to be inactive antiproliferative prodrugs which efficiently regained their cytotoxic efficacy following entrance into cancerous cells. The antiproliferative efficacy of these conjugates was studied in cancerous cell lines of brain glioma origin, to complement another activity of our laboratory aiming to transfer chemotherapeutically active macromolecules from the blood to the brain via the blood–brain barrier (BBB) for treating major brain disorders such as glioblastoma multiforme [14,15,16,17].

The steps involved in preparing the appropriate BSA–MTX conjugates included the application of a chemical procedure enabling linkage of a variety of amino-containing nucleophiles (the spacers) to MTX with the preservation of a significant level of the DHFR-inhibitory potencies (Figure 1, Table 1, Scheme 1); the linkage of these MTX–spacer molecules to the single cysteinyl moiety of BSA, for obtaining monomodified analogues which preserve the native three-dimensional structure of the protein (Scheme 2); determining the DHFR inhibitory potency of these analogues in the cell-free state (Table 2); and studying their cytotoxic potencies in two types of glioma cell lines, one of which is insensitive to MTX (Figure 4A,B).

Of equal interest was to determine the efficacy of those glioma cell lines to regenerate the antiproliferative potencies of albumin–MTX analogues which lost their native three-dimensional configurations (Figure 5A,B).

The superiority of the BSA–MTX conjugates over that of MTX in the insensitive human glioma cell line (Figure 4A) is of profound interest to us. In theory, this superiority might be simply due to impaired MTX-transport into cells [30] which should not affect the albumin–MTX analogues, as they enter cells by the pathway of albumin-mediated endocytosis [31]. It should be noted, however, that this glioma cell line is only partially resistant to MTX (Figure 3); therefore, aberrations, downstream to MTX entry, such as the decrease in thymidylate synthase activity [32], a moderate elevation in the intracellular level of DHFR [33], or a decrease in the binding affinity of MTX to DHFR [23], might take place as well. Those three parameters were documented to be affected to the extent of 1.5- to 3-fold in a variety of other tumorigenic cells that were desensitized to MTX [34].

Although we assumed a priori that the inclusion of a disulfide bond in the spacer connecting MTX to the protein would be significantly beneficial, this assumption was found to be incorrect. Both BSA–S–S–MTX and BSA–(CH_2_)_6_–MTX showed essentially equipotent antiproliferative activity, both in the MTX-sensitive and the MTX-insensitive glioma cell lines (Figure 4), even though glutathione reactivated the conjugate in the cell-free state (Figure 2). It therefore appears that the internalized conjugate is not in contact at all with the cytosolic glutathione. We are currently investigating whether this finding is generally valid for endocytosed-disulfide-containing protein–toxin conjugates.

**Scheme 3:**
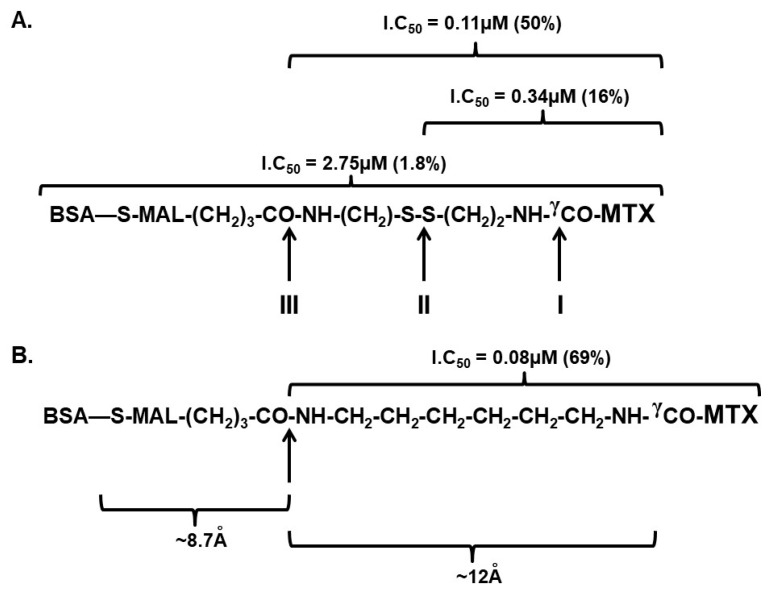
Schematic presentation of the putative endogenously cleaved sites in the BSA–MTX conjugates studied. (**A**) BSA-S-S-MTX; Shown in parenthesis the inhibitory potencies of DHFR by the whole conjugate (I.C_50_ = 2.75 µM) and the inhibitory potencies of the two potentially cleaved MTX containing fragments (I.C_50_ = 0.34 µM and 0.11 µM respectively). (**B**) BSA-(CH_2_)_6_–MTX; Shown in upper parenthesis, the inhibitory potency of DHFR by the potentially cleaved MTX containing fragment (I.C_50_ = 0.08 µM, 69 % the efficacy of non-modified MTX).

Scheme 3 combines our cell-free findings to those found in the cancerous cell lines. BSA–S–S–MTX is a prodrug conjugate having only 1.8% (IC_50_ = 2.75 μM) the inhibitory potency of MTX toward DHFR (Table 2). In theory, the spacer arm connecting MTX to the protein can be endogenously cleaved at three sites (marked I, II, and III in the scheme). Site I, the peptide bond linking MTX via the γ-carboxylate moiety to the spacer, appeared quite uncleavable due to steric hindrance, as previously documented [8]. Site II (the disulfide bond) appears to be negligibly cleaved endogenously as well. BSA–(CH_2_)_6_–MTX, which lacks this disulfide bond_,_ shows equipotent potency as BSA–S–S–MTX (Figure 4). Thus, the peptide bond at site III appeared to be the one which is predominantly cleaved endogenously in the tumorigenic cell lines yielding MTX–CO^γ^NH–(CH_2_)_6_–NH_2_, an MTX derivative which preserves 69% (IC_50_ = 0.08 μM) of the DHFR-inhibitory potency of the native folate antagonist (Table 1). Thus, a simple spacer arm connecting MTX to albumin, made of ethylene-chain, and containing a single peptide bond-distal from the protein backbone by about 8.7 Å, and apart from the covalently linked MTX by about 12 Å, appears most appropriate to bridge cysteine 34 of albumin to MTX (Scheme 3B) for obtaining endogenously-reactivatable prodrug conjugate in the cancerous cell lines studied here. More complicated spacer arms, such as that obtained in our BSA–G–S–S–G–MTX analogue (Scheme 2) appears less promising. It showed low antiproliferative efficacy in the cancer cell lines (unpublished results). These findings also substantiate the poor endogenous efficacy of the tumorigenic cell lines to facilitate glutathione- or cysteine-dependent chemical reduction. Both GSH and cysteine are highly effective in reactivating the DHFR inhibitory potency of this analogue in the cell-free state (Figure 2).

Interestingly, thiosuccinimide linkages are now known to be less robust than once thought, and may undergo maleimide–thiol exchange, for example, by cysteine or GSH present in the external medium [35,36]. This may constitute a drawback to protein–drug conjugates if they undergo such maleimide–thiol exchange. We studied whether such exchange takes place with BSA–S–MAL–(CH_2_)_3_–CONH–MTX, which was incubated for a prolonged period at pH 7.3, 37 °C with high concentrations of reduced glutathione (GSH). We found that such exchange does not occur with this conjugate (Table 3). Cysteine 34 of albumin is located in a deep hydrophobic crevice of 10–12 Å [37]. It therefore seems that the maleimide linked to this cysteine is buried within this crevice, and not exposed to this potential exchange.

Although not in the framework of this study, it appears to us that replacement of MTX therapy by albumin–MTX prodrug therapy is expected to minimize the acquired resistance to MTX which develops upon MTX treatment in cancerous cells. Such analogues that introduce MTX to the cell by an alternative pathway may therefore leave folate carrier free for folic acid entry. Endogenous albumin–MTX cannot be converted to MTX–glutamate or to MTX–polyglutamate by folylpolyglutamate synthetase—an event that prolongs the lifetime of MTX by entrapment [38]. With albumin–MTX, this prolongation is not required, and the induction of desensitization by decreasing the synthesis of MTX–polyglutamate for shortening lifetime of endogenous MTX becomes irrelevant. Dihydrofolate reductase (DHFR) is inhibited by nonmodified MTX at extremely low concentration [39], restricting the inhibitory effect of DHFR by albumin–MTX conjugate, solely to the folic acid-dependent cascade. Undesirable inhibition of RNA and protein synthesis by MTX might be avoided. Inhibition of DHFR per se can still lead to acquired resistance to MTX by increasing DHFR content and/or decrease thymidylate synthase activity. However, such albumin–MTX prodrug analogues preserve low level of active MTX in a continuous fashion, both externally and endogenously following permeation into the cells. This may prevent overdosing of MTX and the consequent side effects.

We have previously found that MTX–amino compounds, such as MTX–CONH–(CH_2_)_6_–NH_2_, covalently linked to carboxylate moieties, such as PEG40–COOH, undergo unusual MTX-dependent acid-dependent peptide bond cleavage, catalyzed by the α-carboxylate moiety of MTX [40]. Unlike what we thought before, this peptide bond is not cleaved in BSA–(CH_2_)_6_–MTX at neutral pH value (Table 3). This is most likely due to the fact that this peptide bond is well located within the 10–12 Å hydrophobic crevice following the covalent linkage to cysteine 34 of BSA. This cleavage, however, expects to take place in an accelerated rate intracellularly due to either degradation of the conjugate and/or by the acidic pH of the lysosome, elevating the reactivation efficacy of those pro-drug conjugates in an intracellular fashion.

In conclusion, the following three conditions appear to be required to engineer the appropriate albumin–MTX prodrug analogues in the future: (i) MTX should be linked to the amino-containing spacer via its γ carboxylate moiety; (ii) the spacer should contain a readily cleavable bond in close proximity to the MTX; (iii) in agreement with previous studies [6,41], the conjugated albumins must preserve their native structure for undergoing extravasation by malignant tissues. As MTX is a drug used also in noncancerous application, such as arthritis, for example, the suggested binding technology might be used also for conjugating it to other macromolecules, such as anti-inflammatory monoclonal antibodies, to increase treatment efficacy and tolerance and to reduce side effects.

## Figures and Tables

**Table 1:** Dihydrofolate reductase inhibitory potencies of methotrexate-amino-containing derivatives.

Derivative Designation	Abbreviated Structure	IC_50_ nM	% Inhibition relative to MTX
Methotrexate	MTX	55 ± 4 ^(a)^	100
MTX–hexylamine	MTX–CONH–(CH_2_)_6_–NH_2_	80 ± 4	69
MTX–dicystamine	MTX–CONH–(CH_2_)_2_–S–S–(CH_2_)_2_–NH_2_	110 ± 12	50
MTX–cystamine	MTX–CONH–(CH_2_)_2_–SH	340 ± 11	16.2
MTX–TAT–peptide	MTX–AYGRKKRRQRRR	1000 ± 200	5.5
HSA–(MTX)_40_	HSA–(NH–CO–MTX)_40_	2100 ± 150	2.6

^(a)^ Determined under the experimental conditions specified in the legend to Figure 1.

**Table 2:** Dihydrofolate reductase inhibitory potencies of BSA–MTX analogues. Reactivation by dithioerythritol (DTT).

Derivative Designation	DHFR Inhibitory Potency (IC_50_, nM)	% Activity Relative to MTX	Fold Reactivation by DTT
BSA–(CH_2_)_6_–MTX	790 ± 30	7.0	
BSA–S–S–MTX	2750 ± 200	2.0	
BSA–S–S–MTX + 2 mM DTT	390 ± 30	14.1	7.05
BSA–GSSG–MTX	1750 ± 120	3.14	
BSA–GSSG–MTX + 2 mM DTT	340 ± 20	16.2	5.16

**Table 3:** Lack of maleimide–thiol exchange of BSA–S–MAL–(CH_2_)_3_–CONH–MTX^(a)^ following treatment with reduced glutathione (GSH).

Treatment	Absorbance at 372 nm per mg Protein
Control	0.479 ± 0.03
GSH ^(a)^	0.487 ± 0.03

^(a)^ BSA–S–MAL–(CH_2_)_3_–CONH–MTX (0.2 mM) dissolved in 1.0 mL 0.1 M HEPES, pH 7.3, and transferred to a tube containing 7.5 mg GSH corresponding to 24 µM which are 100 molar excess over the conjugate. The solution was incubated for 7 h at 37 °C, followed by extensive dialysis and lyophilization.

## Data Availability

The data that support the findings of this study are available from the corresponding authors, upon reasonable request.

## Abbreviations

BSA	bovine serum albumin
MTX	methotrexate
DHF	dihydrofolic acid
DHFR	dihydrofolate reductase
MAL–(CH_2_)_3_–COOSu	maleimidopropionic acid-*N*-hydroxysuccinimide ester
GSSG	oxidized glutathione
BSA–S–S–MTX	BSA–S–MAL–(CH_2_)_3_–CONH–(CH_2_)_2_–S–S–(CH_2_)_2_–NH**^γ^**CO–MTX
BSA–GSSG–MTX	BSA–S–MAL– (CH_2_)_3_–CONH–GSSG–NH**^γ^**CO–MTX
BSA–(CH_2_)_6_–MTX	BSA–S–MAL–(CH_2_)_3_–CONH–(CH_2_)_6_–NH**^γ^**CO–MTX
MALDI-TOF	matrix-assisted laser desorption ionization time of flight
ESMS	electrospray single quadruple mass spectroscopy
DCC	*N,N*′ dicyclohexylcarbodiimide
GSH	reduced glutathione
DMF	dimethylformamide
DIPEA	*N,N*′ diisopropylethylamine
